# Infective endocarditis due to *C. fetus*: an opportunistic infection in a patient with AIDS

**DOI:** 10.1128/asmcr.00102-25

**Published:** 2025-09-10

**Authors:** Cole T. Bredehoeft, Ryan D. Carroll

**Affiliations:** 1 Division of Infectious Diseases, Department of Internal Medicine, College of Medicine, The Ohio State University Wexner Medical Center 12306, Columbus, Ohio, USA; Rush University Medical Center, Chicago, Illinois, USA

**Keywords:** infective endocarditis, *Campylobacter fetus*, human immunodeficiency virus (HIV), acquired immunodeficiency syndrome (AIDS), opportunistic infection

## Abstract

**Background:**

*Campylobacter fetus* is a rare zoonotic cause of infective endocarditis (IE) that occurs most commonly in persons with predisposing conditions, such as immunodeficiencies and/or structural heart disease.

**Case Summary:**

This case highlights native tricuspid valve IE due to *C. fetus* occurring in a patient with concomitant diagnosis of acquired immunodeficiency syndrome (AIDS) treated with combination beta-lactam antimicrobial therapy without immediate surgical candidacy following initial presentation with prolonged constitutional symptoms.

**Conclusion:**

Patients with AIDS are at-risk for opportunistic infections, including those that are less commonly investigated and potentially without obvious epidemiologic risk factors, such as *C. fetus*. However, despite being a fastidious organism, *C. fetus* bacteremia can be diagnosed using widely available blood culture methods. Treatment requires rapid initiation of effective antimicrobial therapy and surgical evaluation in most cases of IE.

## INTRODUCTION

*Campylobacter fetus* is a gram-negative, non-spore-forming, spiral-shaped bacterium that is composed of two humanly relevant subspecies, including *C. fetus* subspecies *fetus* and *C. fetus* subspecies *veneralis*, with both typically being found in the gastrointestinal tract of livestock (1, 2). Nonetheless, human infections have been described with a variety of manifestations ranging from acute gastroenteritis to systemic illnesses, including meningitis, endocarditis, and perinatal infections, including as a cause of abortion, most commonly due to subspecies *fetus* (1, 3). These systemic or disseminated infections tend to occur in human hosts with an underlying predisposing condition, including immunodeficiency and preexisting valvular abnormalities predisposing to infective endocarditis (IE) (1). IE due to *Campylobacter* has been rarely described, though a recent review describing 26 patients between 1966 and 2019 highlighted the propensity of *C. fetus* rather than *C. jejuni* to cause IE (2). Here we describe a case of *C. fetus* native tricuspid valve IE in a patient concomitantly diagnosed with acquired immunodeficiency syndrome followed by development of Kaposi sarcoma (KS).

## CASE PRESENTATION

A 51-year-old male without any notable past medical history presented to the emergency department with a 1-year history of chills and 4-week history of progressive fatigue accompanied by fever, weight loss of 20–30 lbs, and productive cough. The examination was notable for a malnourished and underweight appearance. Laboratory findings were notable for lymphopenia (absolute lymphocyte count 0.17 K/mL [0.83–3.57 K/mL]), microcytic anemia (hemoglobin 7.1 g/dL [13.4–16.8 g/dL], mean corpuscular volume 85.8 fL [79.0–94.5 fL]), and mild hyponatremia (sodium 131 mmol/L [135–145 mmol/L]). The chest radiograph was without acute cardiopulmonary abnormalities, and computed tomography (CT) of the abdomen and pelvis with contrast was without acute processes. He was then admitted for further evaluation of weight loss.

During his evaluation, he requested to be screened for sexually transmitted infections, which included screening for human immunodeficiency virus (HIV), which confirmed HIV-1 infection. Subsequent HIV-1 viral load was 3,918 copies/mL (log 3.59), and CD4 lymphocyte count was 12 ABS/mm^3^ (266–2,313 ABS/mm^3^), or 7.3% (32.0–62.0%). Infectious Diseases was consulted, and further history elicited that he had been previously diagnosed with HIV, though he was not engaged in care and had never received antiretroviral therapy (ART). Evaluation for opportunistic infections, including *Cryptococcus*, was pursued and was unrevealing.

He was started on bictegravir/emtricitabine/tenofovir alafenamide and trimethoprim–sulfamethoxazole prophylaxis on day 3 of hospitalization. On day 4 of hospitalization, blood cultures collected on admission resulted in *C. fetus* with growth in four of four bottles collected (two aerobic and two anaerobic) within 48–72 h. Prior to identification by MALDI-TOF, the blood culture instrument detected growth on the initial culture with concern for false positivity due to lack of organisms identified on Gram stain. However, after 48 h of incubation, there was a “haze or scum” on agar plates following ambient air incubation at 37°C with 7% CO_2_. After identification, he was started on combination therapy for *C. fetus* bacteremia with intravenous imipenem–cilastatin 500 mg every 6 h and oral amoxicillin–clavulanate 875–125 mg every 12 h. Repeat blood cultures were without recurrent isolation following initiation of antibiotic therapy.

**Fig 1:**
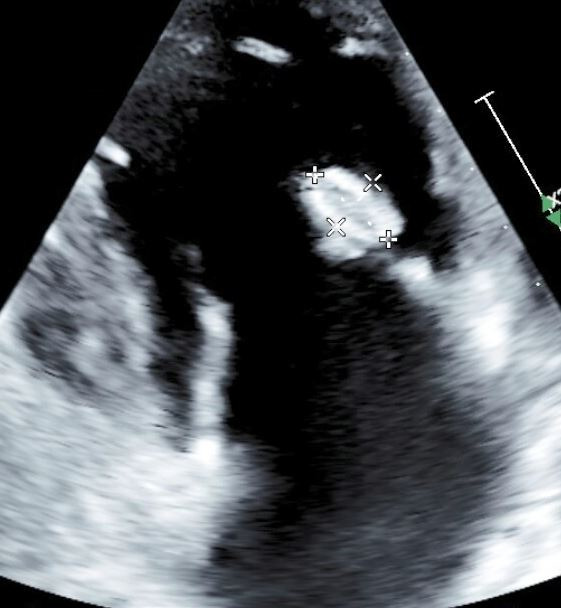
TTE demonstrating native tricuspid valve vegetation.

Transthoracic echocardiogram (TTE) was obtained and revealed a 1.5 × 1.1 cm oval, mobile mass with protruding elements attached to the tricuspid valve with mild tricuspid valve regurgitation (Fig. 1). Given surgical indications, he was evaluated by cardiac surgery, who recommended deferment of valve replacement with continued medical therapy with a plan for follow-up, and he was subsequently discharged on imipenem–cilastatin and amoxicillin–clavulanate to complete a 6-week course for IE in addition to ART and prophylaxis.

Susceptibility data were later returned from the performing reference laboratory, as seen in Table 1. Given previous clinical improvement and blood culture clearance, no changes were made to this treatment regimen based on this data.

**TABLE 1:** Antimicrobial susceptibility of *C. fetus*

Antimicrobial	MIC (mcg/mL)
Ciprofloxacin	>2
Erythromycin	2
Gentamicin	1
Tetracycline	2

His treatment course was complicated by a recurrent admission two weeks following discharge with intermittent fevers, inability to tolerate oral intake, fatigue, and myalgias with laboratory studies notable for anemia and thrombocytopenia. At that time, CD4 lymphocyte count had risen to 230 ABS/mm^3^ (266–2,313 ABS/mm^3^) or 14.1% (32.0–62.0%). Hematologic abnormalities were attributed to trimethoprim–sulfamethoxazole with transition to atovaquone for prophylaxis, and he was treated for immune reconstitution inflammatory syndrome with a corticosteroid taper, which resulted in improvement in fevers and symptoms with subsequent discharge.

One month following hospitalization, he was evaluated in the Infectious Diseases clinic. At that time, he reported progressively worsening shortness of breath with productive cough of white, foamy sputum as well as fevers and night sweats. The examination was notable for a toxic-appearing male, respiratory distress with tachypnea and accessory muscle use, and cervical lymphadenopathy. Given his clinical status, he was transferred to the emergency department.

Upon recurrent admission, a CT chest with contrast was obtained, which demonstrated left hilar nodular opacity likely representing an enlarged lymph node along with bilateral hilar, mediastinal, axillary, and supraclavicular lymphadenopathy. Repeat TTE demonstrated calcification of tricuspid valve vegetation with progressive tricuspid valve regurgitation. He was evaluated by Infectious Diseases with subsequent recommendations for evaluation of bacterial, mycobacterial, fungal, and viral etiologies, which were unrevealing. Notably, the repeat CD4 lymphocyte count was 16 ABS/mm^3^ (266–2,313 ABS/mm^3^), or 9.2% (32.0–62.0%) and HIV viral load was undetectable on admission.

His clinical status improved with supportive care, and on day 5 of hospitalization, he underwent an excisional inguinal lymph node biopsy. The excised lymph node was sent for histopathological evaluation as well as bacterial, mycobacterial, and fungal cultures. At that time, quantitative serum for Epstein-Barr virus (EBV) and human herpesvirus-8 (HHV-8) polymerase chain reactions resulted in 19,849 IU/mL (<1,000 IU/mL) and >10,000,000 copies/mL, respectively. Histopathology of the excised lymph node demonstrated spindle cells with immunohistochemical stains for HHV-8 consistent with KS. There were scattered cells stained with EBV; however, there was no evidence of abnormal B- or T-lymphocytes on flow cytometry. Subsequent positron emission tomography demonstrated innumerable intensely avid supra- and infra-diaphragmatic lymph nodes as well as bilateral lung nodules. He was started on liposomal doxorubicin with treatment ongoing at this time, and he is being considered for elective valve repair in the future. He has remained virally suppressed on bictegravir/emtricitabine/tenofovir alafenamide.

## DISCUSSION

*C. fetus* is a zoonotic pathogen that has been found across the globe with livestock serving as reservoirs with human infections due to ingestion of meat products (1, 2). Nearly all human infections are due to subspecies *fetus*, which is considered an opportunistic pathogen that has a propensity to cause serious systemic infections in patients with underlying immunodeficiency (i.e., HIV, hematologic malignancies), cardiac valvular abnormalities, liver disease, or implanted devices, though it has been seen in otherwise healthy older individuals or during pregnancy (1, 3).

After ingestion, *C. fetus* adheres to the mucosal surfaces due to its spiral shape and flagellum-mediated motility allowing for both trans- and paracellular epithelial migration (3). Cytolethal distending toxins then mediate DNA damage leading to epithelial cell swelling, resulting in barrier disruption allowing for propagation of infection (3). This pathogen can then cause relapsing or persistent infections due to the ability to survive while evading the host immune response due to a chromosomally encoded surface layer (S-layer), which comprises a capsule structure resistant to mucosal phagocytosis as well as complement-mediated killing with further antigenic variation allowing for evasion of the host innate immune response (1, 3).

The diagnosis of *C. fetus* infections and their complications proves difficult as it requires provider awareness of the organism and its complications. This fastidious organism requires a microaerobic environment for growth, though modern blood culture methods allow for growth without use of selective media, as was seen in this patient (1, 4). Identification of this uncommon organism should prompt clinicians to consider invasive complications such as endovascular infections, especially in susceptible hosts such as those with immunodeficiencies. Those with IE due to *Campylobacter* present with a myriad of symptoms, including nonspecific constitutional symptoms as well as sequelae from valvular destruction and embolic phenomena (2). With regard to IE, the aortic valve is most frequently involved followed by the mitral valve; however, this patient had tricuspid valve involvement (2, 4, 5).

Treatment of IE due to *Campylobacter* is complex and typically requires a combination of antimicrobial therapy and surgical intervention; however, mortality remains quite high (2, 5, 6). In a review of IE due to *Campylobacter*, most patients received combinations of beta-lactams, aminoglycosides, and/or macrolides, as resistance to tetracyclines and quinolones has been described not infrequently (2). In a recent French study, including 38 patients with vascular infections and 13 patients with IE, none of the strains were found to be resistant to amoxicillin–clavulanate or imipenem, as utilized in this case; however, there were high rates of fluoroquinolone and tetracycline resistance exceeding 20% (4). Relapse has been observed with delayed initiation of effective antimicrobial therapy in the setting of its aforementioned evasive S-layer (1, 4). Clinical Laboratory Standards Institute (CLSI) and European Committee on Antimicrobial Susceptibility Testing (EUCAST) breakpoints do not currently exist for this pathogen and thus, clinicians must rely on empiric choices based on patient-specific factors (7).

*Campylobacter* cardiovascular infections, including IE, are rare but are correlated with high mortality, particularly in those with predisposing medical conditions. This pathogen should be considered in those at higher risk of systemic infection and associated complications who present with gastroenteritis and/or preceding gastrointestinal symptoms with concern for manifestations of IE. Persistent and relapsed infections occur with improvement in outcomes with early initiation of effective antimicrobial therapy and surgical evaluation along with addressing underlying predisposing conditions, such as ART for immune reconstitution in this patient’s case.

## Data Availability

Data sharing is not applicable to this article as no new data were created or analyzed in this study.
